# Is it time to rethink the relationship between adipose inflammation and insulin resistance?

**DOI:** 10.1172/JCI184663

**Published:** 2024-09-03

**Authors:** Evan D. Rosen, Shingo Kajimura

**Affiliations:** 1 Division of Endocrinology, Diabetes, and Metabolism, Beth Israel Deaconess Medical Center, Boston, Massachusetts, USA.; 2 Harvard Medical School, Boston, Massachusetts, USA.; 3 Broad Institute of MIT and Harvard, Cambridge, Massachusetts, USA.; 4 Howard Hughes Medical Institute, Chevy Chase, Maryland, USA.

## Uncovering inflammation in adipose tissue

**Figure 1:**
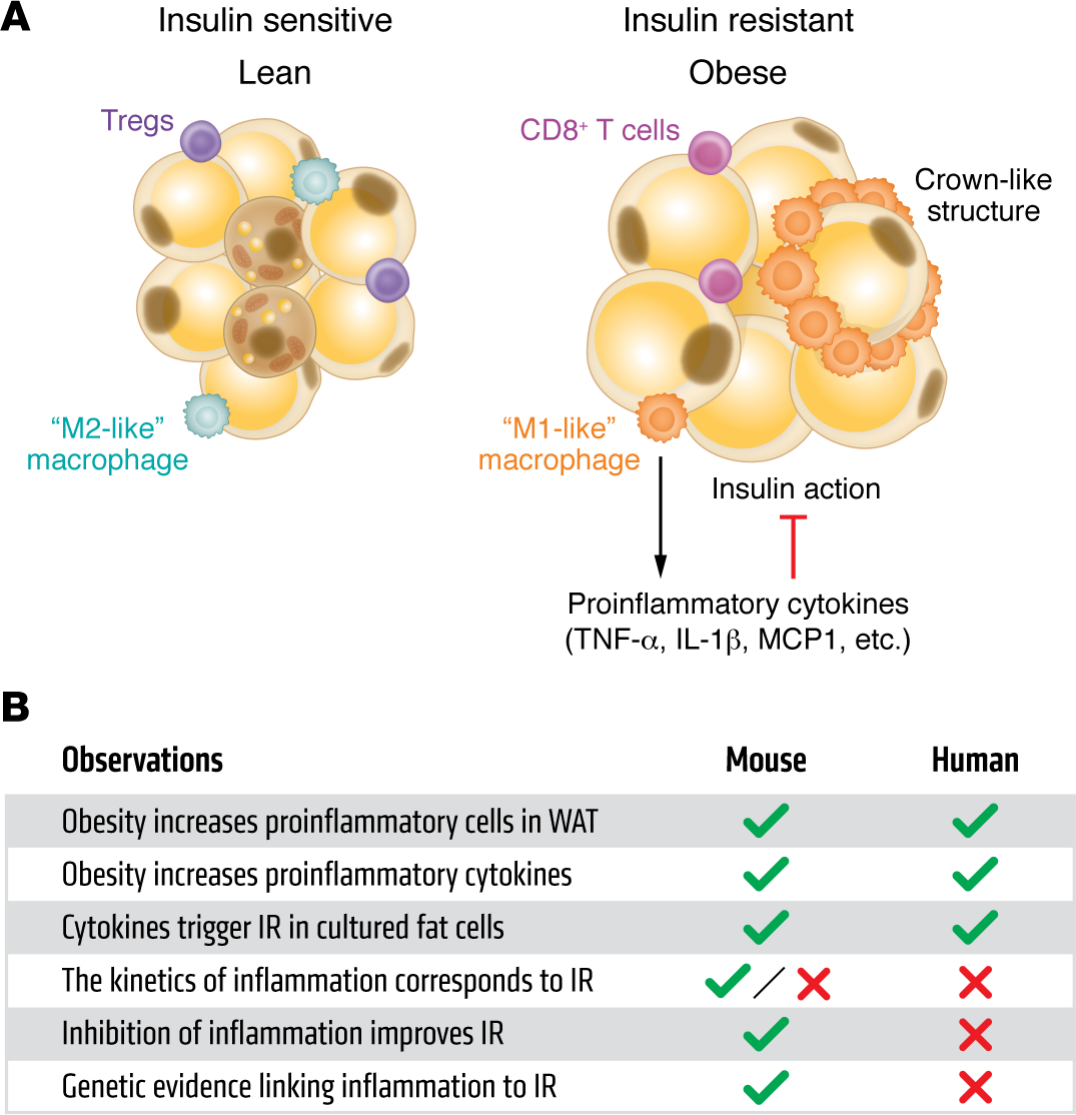
Current paradigm of the relationship of adipose inflammation to insulin resistance. (**A**) In the setting of obesity, a generally antiinflammatory immune milieu is replaced by proinflammatory immune cells, like “M1-like” macrophages, CD8^^+^^ T cells, and others. These cells secrete cytokines that reduce insulin action in the adipocyte and, eventually, systemically. (**B**) Critical observations that support the paradigm in **A** and whether they have been shown in mice and humans. IR, insulin resistance.

The association between obesity and diabetes has been recognized since ancient times; as early as the fifth century BCE, the Indian physician Sushruta linked diabetes in wealthy people to excessive consumption of grains and sweets (1). A major breakthrough emerged in the early 1990s when Hotamisligil and Spiegelman demonstrated that the expression of TNF-α was elevated in the adipose tissue of obese rodents and that neutralization of this inflammatory cytokine could restore insulin sensitivity (2). Subsequent studies in the *JCI* by the same group showed that TNF-α impairs the tyrosine kinase activity of the insulin receptor and that humans with obesity and insulin-resistant humans display elevated expression of adipose TNF-α (3, 4). It was assumed that the source of TNF-α and other cytokines in obesity was the adipocyte itself, but it was noted that pure cultures of adipocytes in vitro do not express TNF-α. This conundrum was resolved in 2003, with the publication in this journal of two seminal papers showing that the macrophage content of adipose tissue in mice goes up dramatically in both diet-induced and genetic obesity and that these proinflammatory cells are the source of TNF-α and many other cytokines (5, 6) (Figure 1). Later studies expanded on this finding, showing that a wide portfolio of innate and adaptive immune cells exists in adipose tissue, including macrophages and monocytes of several types, B and T lymphocytes, neutrophils, eosinophils, basophils, mast cells, dendritic and natural killer cells, and innate lymphoid cells. Moreover, the relative numbers and activity of “proinflammatory” immune cells are profoundly induced in obesity, creating a state of so-called “meta-inflammation,” which drives insulin resistance (reviewed in ref. 7).

This idea is supported by numerous studies in mice: genetic or pharmacologic manipulation of specific proinflammatory immune cell populations restores insulin sensitivity in obese mice, as does blockade or deletion of various chemokines, cytokines, and their receptors. Furthermore, a wide variety of interrelated effector pathways have been implicated as mechanistic links between inflammation and insulin resistance, such as oxidative stress, ER stress, mitochondrial dysfunction, and direct modification of insulin signaling components (8). Interestingly, while some studies have demonstrated that macrophage infiltration and inflammation occur prior to the onset of insulin resistance in mice (which would be required for inflammation to be considered causal) (5), others have questioned this temporal relationship and have even suggested that insulin resistance causes inflammation and not vice versa (9). A recent study also showed that adipose tissue inflammation persisted even after weight loss and improved glycemic control in mice (10).

## Does inflammation drive insulin resistance in humans?

Humans show many correlates of the key findings from mouse studies: adipose tissue of individuals with obesity contains a significantly higher proportion of proinflammatory immune cells and reduced levels of antiinflammatory populations. Individuals with obesity display elevated cytokine levels, both locally within fat and systemically, and there is a positive correlation between indices of white adipose tissue (WAT) inflammation and insulin resistance and/or dysglycemia (7). Furthermore, at least some cytokines (e.g., IL-1β) can promote insulin resistance in isolated human adipocytes (11). These parallels to the murine situation have led to the current consensus in the field that enhanced intra-adipose and subsequent systemic inflammation provoke insulin resistance in humans.

Despite the compelling preclinical data just described, there is mounting evidence that this paradigm may not reflect the full picture of what is happening in human obesity and type 2 diabetes (T2D). In our view, three lines of evidence from physiologic, genetic, and pharmacologic studies are inconsistent with the notion that adipose inflammation is a causal driver of human insulin resistance and T2D. First, immune infiltration and insulin resistance can be uncoupled in humans, particularly in the context of weight loss. For example, 5%–10% weight loss by diet and exercise significantly improved clinical measures of insulin sensitivity without affecting adipose macrophage content and tissue or systemic cytokine levels (12, 13). In a study of individuals whose metabolic parameters were improved after bariatric surgery, adipose macrophage content and inflammation were reduced at 3 months after surgery; however, the temporal correlation to the metabolic improvement is not clear (14). Second, genome-wide association studies (GWAS) for T2D and other traits associated with insulin sensitivity have identified genes associated with adipocyte differentiation (e.g., *PPARG*) and insulin signaling (e.g., *IRS1*, *SLC2A4*), in addition to many genes without an immediately apparent connection to insulin action. What has not shown up in these GWAS are genes associated with inflammation, especially the genes that have been best characterized as metabolically relevant in the mouse, such as *TNFA*, *IL1B*, and *CCL2*. A potentially trivial explanation for this could be insufficient variation around these loci to show up as hits in even the largest GWAS. However, this is not the case, as these genes are well represented in GWAS for rheumatoid arthritis, lupus, and other inflammatory diseases.

Third, powerful antiinflammatory drugs have been developed for use in human immune diseases, including agents that target TNF-α (e.g., adalimumab, infliximab, and etanercept), IL-1 (e.g., anakinra), and IL-6 (e.g., sarilumab). Anecdotal evidence suggests that there is no need to adjust antidiabetic medications in patients receiving these agents. In addition, some of these agents have been formally tested for metabolic benefit, with uninspiring results. Improved glycemia has been reported with anakinra, but this effect was associated with improved β cell function not insulin sensitivity (15). Similarly, no effect on homeostatic model assessment of insulin resistance was noted with diacerein, which blocks both TNF-α and IL-1β, although the diacerein group displayed a modest reduction in mean HbA1c level (16). Etanercept did not improve insulin sensitivity, even though it effectively reduced the inflammatory marker, C-reactive protein (17). Some studies with high-dose salsalate showed beneficial effects on glycemia; however, these actions are likely attributable to activation of AMP kinase rather than suppression of inflammation (18).

## Outstanding questions in the field

These observations regarding inflammation and insulin resistance in humans raise several critical questions. Could differences between humans and mice account for discrepancies in their metabolic response to immune perturbations? While simple species differences would seem to be an unsatisfactory answer on the surface, there is precedent in the example of sepsis. Mice suffer from septic shock in a manner analogous to humans, but the details differ quite significantly; humans and mice do not have the same sensitivities to mediators of sepsis, and the cytokine profiles involved are species specific (19). It is possible that inflammation does promote insulin resistance in human obesity, but we may have been focusing on the wrong immune effectors and pathways. For example, some loci identified in T2D GWAS (e.g., *MAP3K3*, *CFB*, *C2*, and various HLA genes) are associated with genes with roles in the immune system. If these alterative inflammatory mediators are important in human insulin resistance, then it would not be surprising that drugs targeting TNF-α and other “classical” cytokines would be inefficacious in reversing insulin resistance.

Perhaps adipose tissue inflammation is not the cause of T2D in most people, but could it drive insulin resistance in a select subgroup? There is some evidence for this; in a small clinical trial, Oral, Saltiel, and colleagues showed that an inhibitor of the proinflammatory kinases TBK1 and IKKε (amlexanox) improved insulin sensitivity in a subset of patients with T2D. This subgroup was characterized by higher inflammatory gene expression in their adipose tissue at baseline (20). Notably, however, clustering of large numbers of patients with T2D using either genetic variants or clinical phenotypes has so far failed to identify a specific subgroup with inflammation as a defining feature (21, 22).

What is the role, if any, of proinflammatory immune cells in adipose tissue in obesity? In other words, if they are not driving insulin resistance, what are the proinflammatory immune cells doing? A highly salient observation was made by Cinti, Obin, and colleagues that adipose tissue macrophages in obesity are not distributed evenly throughout the fat pad, but they are instead clustered around dead or dying adipocytes (23). This finding is consistent with the role of macrophages in “remodeling” the fat pad, in part by taking up the lipid released by dying adipocytes. Many macrophage subpopulations have also been identified in WAT with important roles in vascularization, innervation, and lipid homeostasis (reviewed in ref. 24). This remodeling function may explain why macrophages and other immune cells accumulate in WAT after weight gain.

## Concluding remarks

There appears to be a direct, causal link between adipose inflammation and insulin resistance in mice. In humans, we need more information. First, we can expect new data soon that will shed light on this issue. Emerging single-cell studies will provide an opportunity to revisit and refine the temporal relationship between adipose inflammation and obesity-associated insulin resistance in humans in settings of weight gain and loss. In addition, there are intensive efforts underway to characterize the heterogeneity of T2D in diverse populations at the phenotypic and molecular levels; perhaps an inflammation-driven subpopulation may emerge. Similarly, ongoing work is slowly revealing the precise mechanisms by which common noncoding variation predisposes to T2D — a role for inflammation may yet emerge from these studies. For now, however, we believe that the community should approach the notion that inflammation is the driver of insulin resistance in humans as a hypothesis, rather than a proven fact.

## Version 1 09/03/2024

Electronic publication
